# Novel inflammatory metabolic parameters as predictors of metabolic dysfunction-associated fatty liver disease in people living with HIV receiving antiretroviral therapy: a retrospective cohort study

**DOI:** 10.3389/fpubh.2026.1716876

**Published:** 2026-01-21

**Authors:** Jinjiao Hu, Ye Fang, Suya Ma, Yong Jin

**Affiliations:** 1 Department of Ultrasonic, Ningbo Hospital of Integrated Traditional Chinese and Western Medicine, Ningbo, Zhejiang, China; 2 Health Science Center, Ningbo University, Ningbo, Zhejiang, China; 3 Department of Infectious Diseases, Ningbo Hospital of Integrated Traditional Chinese and Western Medicine, Ningbo, Zhejiang, China

**Keywords:** cohort study, integrase strand transfer inhibitors, metabolic dysfunction-associated fatty liver disease, novel inflammatory metabolic parameters, people living with HIV

## Abstract

**Background:**

Metabolic dysfunction-associated fatty liver disease (MAFLD) has emerged as a major non-communicable comorbidity in people living with HIV (PLWH). The role of inflammatory metabolic parameters—including the lymphocyte-to-high-density lipoprotein cholesterol ratio (LHR), platelet to high-density lipoprotein cholesterol ratio (PHR), and aggregate index of systemic inflammation (AISI)—in predicting MAFLD in PLWH remains unclear. This study aimed to evaluate the prognostic value of inflammatory metabolic parameters for predicting MAFLD in PLWH.

**Methods:**

We conducted a retrospective cohort study of 814 PLWH receiving stable antiretroviral therapy (ART) between 2018 and 2025 at a tertiary care center. Baseline demographic, clinical, and laboratory data were collected. LHR, PHR, and AISI were calculated and categorized into tertiles. The primary outcome was incident MAFLD, diagnosed according to Chinese guidelines. Kaplan–Meier survival analysis, Cox proportional hazards regression, restricted cubic spline (RCS) models, and time-dependent receiver operating characteristic (ROC) curves were applied to assess associations and predictive performance. Sensitivity analyses were performed across subgroups.

**Results:**

During a median follow-up of 2.8 years, 116 participants (14.3%) developed MAFLD, corresponding to an incidence rate of 50.4 per 1,000 person-years. Higher LHR and PHR tertiles were significantly associated with increased MAFLD risk, whereas AISI showed no predictive value. In fully adjusted Cox models, the high LHR tertile remained an independent risk factor (HR = 2.315, 95% CI: 1.208–4.436, *p* = 0.011), while only the middle PHR tertile retained significance. RCS analyses showed no significant non-linear associations for LHR, PHR, or AISI. Time-dependent ROC analyses demonstrated that LHR had the strongest short-term predictive ability (AUC = 0.713 at 1 year), followed by PHR (AUC = 0.644), while AISI consistently performed poorly (AUC < 0.600). Subgroup analyses confirmed the robustness of LHR and PHR associations across demographic, clinical, and metabolic subgroups.

**Conclusion:**

In this large cohort of PLWH on ART, LHR and PHR were independent predictors of incident MAFLD, with LHR demonstrating the strongest and most consistent predictive value. AISI was not associated with MAFLD. LHR and PHR, as simple, low-cost indices derived from routine laboratory tests, may serve as practical tools for identifying high-risk individuals who could benefit from early monitoring and targeted intervention.

## 1 Introduction

Metabolic dysfunction-associated fatty liver disease (MAFLD) has become a major cause of chronic liver disease globally, and its prevalence is increasingly acknowledged in people living with HIV (PLWH) (1). MAFLD is characterized by hepatocellular lipid accumulation and is associated with metabolic comorbidities including obesity, insulin resistance, dyslipidemia, and hypertension (2). Retrospective cohort studies report a prevalence of about 13.9% in PLWH, underscoring a substantial disease burden in this group (3). The progression of MAFLD may lead to hepatic fibrosis, decompensated cirrhosis, and liver-related mortality, and is also linked to extrahepatic comorbidities such as chronic kidney disease, cardiovascular disease, and obstructive sleep apnea (4). In the era of antiretroviral therapy (ART), as opportunistic infections decline, non-communicable diseases such as MAFLD have become key determinants of quality of life and long-term survival in PLWH. Moreover, fatty liver disease is associated with worse outcomes and reduced responses to antiviral therapy in PLWH (5). Despite these implications, PLWH are frequently excluded from large-scale epidemiological studies, restricting knowledge of MAFLD in this population. Compared with the general population, MAFLD pathogenesis in PLWH is more complex, involving chronic low-grade inflammation, immune dysregulation, and the metabolic consequences of long-term ART exposure (6). Against this background, identifying new risk factors for MAFLD in PLWH is crucial for guiding early detection and improving clinical outcomes.

The immune and metabolic systems are both crucial in the development and progression of MAFLD in PLWH. Immunological mechanisms are central to the pathogenesis of nonalcoholic steatohepatitis, with B cells and T cells actively driving hepatic inflammation and fibrogenesis. Specifically, interleukin-17, secreted by CD4 + T and CD8 + T cells, mediates systemic inflammation and promotes the recruitment of inflammatory cells into the liver, thereby playing a key role in MAFLD development (7). At the same time, HIV infection and long-term ART exposure cause profound metabolic alterations, including dyslipidemia, insulin resistance, and adipose tissue redistribution, which synergistically worsen hepatic steatosis and accelerate disease progression (8, 9). Recently, several novel inflammatory metabolic parameters have been proposed to capture the interaction between immune dysregulation and metabolic dysfunction. For example, the lymphocyte-to-high-density lipoprotein cholesterol ratio (LHR), which integrates immune and metabolic status, has been identified as a biomarker of MAFLD severity (10). Similarly, the platelet to high-density lipoprotein cholesterol ratio (PHR), a composite marker of metabolic and coagulation status, has been validated as a reliable indicator for both fatty liver disease and hepatic fibrosis (11). The aggregate index of systemic inflammation (AISI), which integrates neutrophil, platelet, monocyte, and lymphocyte counts, provides a comprehensive measure of the balance between pro-inflammatory and immune-regulatory responses. Studies in hypertensive populations have shown that AISI is strongly associated with MAFLD, suggesting its potential use as a surrogate marker of systemic inflammation–driven metabolic dysfunction (12).

Several inflammatory metabolic parameters are associated with the presence and severity of MAFLD in the general population. However, their relevance to PLWH remains unclear, owing to distinctive pathophysiological features such as chronic immune dysfunction, persistent low-grade inflammation, and long-term metabolic effects of ART. To address these gaps, we conducted a retrospective cohort study to evaluate the prognostic value of multiple inflammatory metabolic parameters for predicting MAFLD in PLWH receiving ART.

## 2 Methods

### 2.1 Study design and population

**Figure 1:**
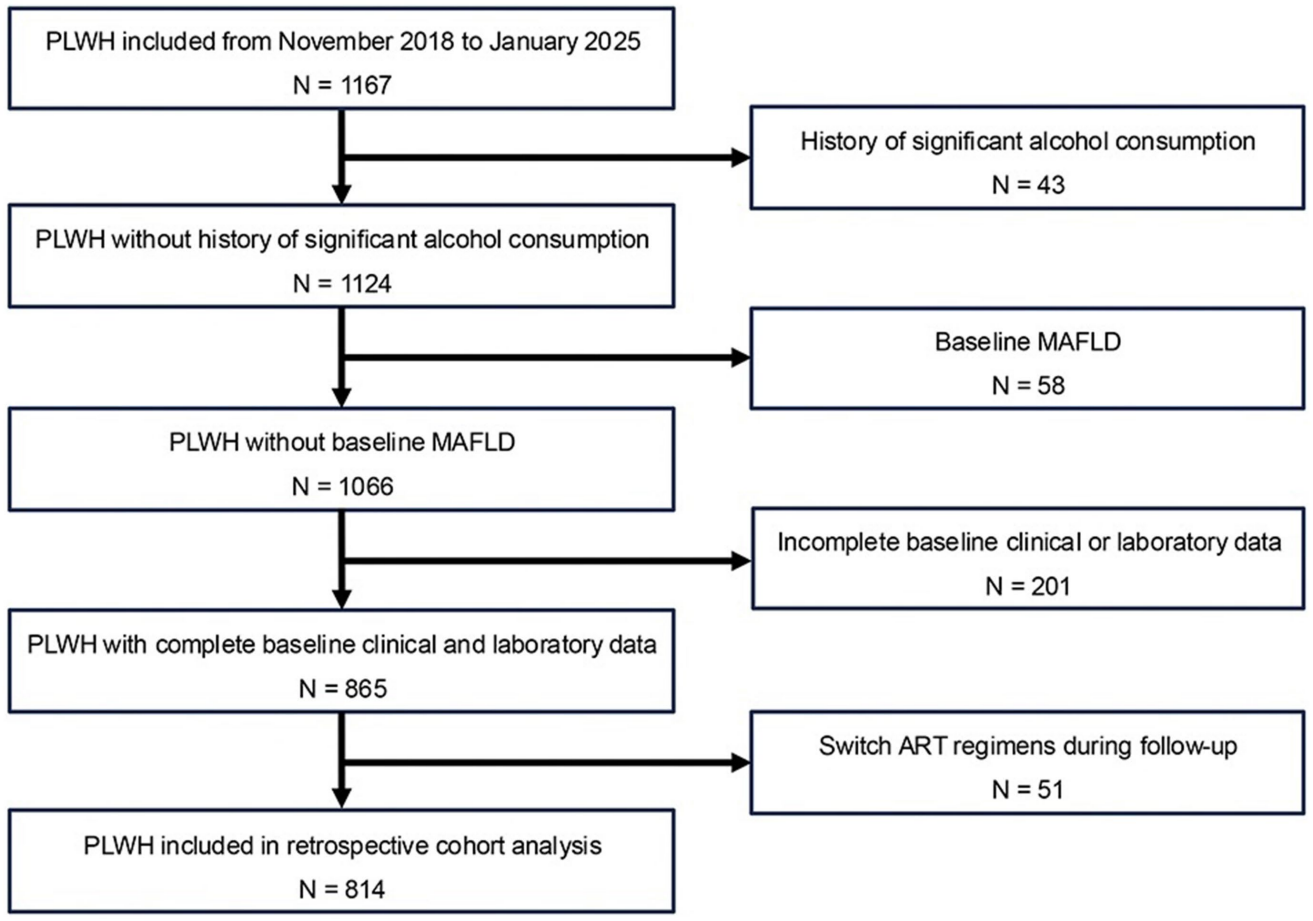
Flowchart showing selection of people living with HIV (PLWH) from November 2018 to January 2025 for retrospective cohort analysis. Initial group of 1167, reduced to 814 through exclusions: 43 for significant alcohol consumption history, 58 with baseline MAFLD, 201 with incomplete baseline data, and 51 who switched ART regimens during follow-up.

This retrospective cohort study was conducted at Ningbo Hospital of Integrated Traditional Chinese and Western Medicine, a tertiary care center providing comprehensive HIV care. Electronic medical records of PLWH receiving stable ART between 2018 and 2025 were reviewed. Eligible participants were adults aged ≥18 years with confirmed HIV infection and complete clinical, laboratory, and imaging data required for assessing hepatic steatosis and inflammatory metabolic parameters. Exclusion criteria included a history of significant alcohol consumption (≥210 g/week for men and ≥140 g/week for women), evidence of other chronic liver diseases (e.g., autoimmune hepatitis, drug-induced liver injury, or hereditary liver diseases), use of hepatotoxic medications unrelated to ART, incomplete baseline clinical or laboratory data, or switching ART regimens during follow-up because of virological failure or other clinical indications. Individuals with baseline MAFLD were also excluded to ensure accurate evaluation of incident cases during the study period. After applying these criteria, 814 PLWH were included in the final analysis. A schematic overview of the study design and participant selection is shown in Figure 1.

### 2.2 Measurement of novel inflammatory metabolic parameters

Novel inflammatory metabolic parameters were measured using data from routine blood tests. Three parameters were calculated for each participant:

1. LHR = lymphocyte count (×10^9^/L) / HDL-C (mmol/L).
2. PHR = platelet count (×10^9^/L) / HDL-C (mmol/L).
3. AISI = (neutrophil count × monocyte count × platelet count) / lymphocyte count, all expressed in ×10^9^/L.

For analysis, each parameter was categorized into tertiles (low, middle, high), with the lowest tertile as the reference group. This categorization enabled evaluation of dose–response relationships between inflammatory metabolic parameters and the risk of MAFLD in PLWH.

### 2.3 Definition of MAFLD

The primary outcome was the incidence of MAFLD. Hepatic steatosis was diagnosed by abdominal ultrasonography performed by experienced radiologists using standardized protocols. According to Chinese guidelines (13), MAFLD was diagnosed when hepatic steatosis was present in combination with at least one metabolic component: (1) overweight / central obesity, defined as body mass index (BMI) ≥ 24 kg/m^2^ or waist circumference (WC) ≥ 90 cm in men and ≥85 cm in women; (2) hypertension, defined as systolic blood pressure ≥130 mmHg, diastolic blood pressure ≥85 mmHg, or current use of antihypertensive drugs; (3) hyperglycemia, defined as fasting blood glucose ≥6.1 mmol/L, postprandial blood glucose ≥7.8 mmol/L, or a prior diagnosis of diabetes mellitus; (4) Elevated triglycerides (TG), defined as fasting serum TG ≥ 1.7 mmol/L or ongoing treatment with lipid-lowering therapy; or (5) reduced high-density lipoprotein cholesterol (HDL-C), defined as ≤1.0 mmol/L in men and ≤1.3 mmol/L in women. All participants underwent routine clinical follow-up approximately every 3 months and were monitored until the first occurrence of MAFLD, as defined above, or until the end of the study period in January 2025.

### 2.4 Assessment of baseline covariates

Baseline data were defined as the information recorded at each patient’s first outpatient visit to our hospital. A comprehensive set of demographic characteristics, medical history, and laboratory measurements was collected. Demographic variables included age, sex, self-reported HIV transmission route (heterosexual contact, men who have sex with men [MSM], or other/unknown), initial ART regimen, height, weight, and WC. BMI was calculated as weight (kg) divided by height squared (m^2^). Initial ART regimens were classified as standardized therapy, consisting of two nucleoside reverse transcriptase inhibitors (NRTIs) plus a third agent: either an integrase strand transfer inhibitor (INSTI), a non-nucleoside reverse transcriptase inhibitor (NNRTI), or a protease inhibitor (PI). In addition, the fixed-dose combination of lamivudine (3TC) and dolutegravir (DTG) was considered an acceptable alternative regimen. Metabolic comorbidities relevant to the diagnosis of MAFLD were recorded, including reduced HDL-C, overweight/central obesity, elevated TG, hyperglycemia, and hypertension. Baseline laboratory measurements included HIV viral load, hemoglobin (HGB), platelet count (PLT), neutrophil count (NEUT), lymphocyte count (LYMPH), monocyte count (MONO), eosinophil count (EO), basophil count (BASO), CD4 T cell count, HDL-C, TG, and fasting or postprandial blood glucose. Baseline HIV viral load values were log₁₀-transformed for normalization and categorized into three groups: viral suppression (<50 copies/mL or undetectable), low viral load (detectable but <3 log₁₀ copies/mL), and high viral load (>3 log₁₀ copies/mL).

### 2.5 Statistical analysis

The Kolmogorov–Smirnov test was used to assess the normality of continuous variables before statistical analyses. Continuous variables were expressed as medians with interquartile ranges (IQRs) and compared using the Mann–Whitney U test or Kruskal–Wallis H test, as appropriate. Categorical variables were summarized as frequencies and percentages and compared using the χ^2^ test or Fisher’s exact test. Kaplan–Meier curves were generated to estimate cumulative incidence of MAFLD, and group differences were assessed with the log-rank test. Associations between novel inflammatory metabolic parameters and incident MAFLD were examined using univariate and multivariate Cox proportional hazards regression, with results reported as hazard ratios (HRs) and 95% confidence intervals (CIs). Three models were constructed: Model 1, unadjusted; Model 2, adjusted for age and sex; and Model 3, further adjusted for ART regimen, reduced HDL-C, overweight/central obesity, elevated TG, hyperglycemia, hypertension, HGB, and CD4 T cell count. Multicollinearity among covariates was assessed using the variance inflation factor (VIF), with values>10 indicating severe multicollinearity. Because of multicollinearity with novel inflammatory metabolic parameters, NEUT, LYMPH, and MONO were excluded from Model 3. Potential non-linear associations between LHR, PHR, and AISI and MAFLD were evaluated using restricted cubic spline (RCS) analyses with four knots to balance smoothness and prevent overfitting. Time-dependent receiver operating characteristic (ROC) curves were used to assess predictive performance of the three parameters for incident MAFLD at 1, 3, and 5 years. Sensitivity analyses were performed to test robustness, including subgroup analyses stratified by sex, age, HIV transmission risk, ART regimen, baseline HIV viral load, and metabolic comorbidities. All analyses were conducted using R version 4.4.2, SPSS version 29.0, and GraphPad Prism version 10.0, with two-tailed *p* < 0.05 considered statistically significant.

### 2.6 Ethics approval and consent to participate

The Institutional Review Board of Ningbo Hospital of Integrated Traditional Chinese and Western Medicine approved this study (2023–050) and adhered to the Helsinki Declaration of 1964, along with its subsequent updates. The requirement for informed consent was waived due to the retrospective study design.

## 3 Results

### 3.1 Baseline characteristics of PLWH

A total of 814 PLWH were included in the analysis (Figure 1). Of these, 703 (86.4%) were male, with a median age of 37 years. The most common HIV transmission route was MSM (46.7%), followed by heterosexual contact (44.7%) and other/unknown routes (8.6%). Regarding ART regimens, most participants received NNRTIs + NRTIs (66.5%) as first-line therapy, followed by INSTIs + NRTIs (25.7%), the fixed-dose combination of 3TC and DTG (4.5%), and PIs + NRTIs (3.3%). With respect to metabolic comorbidities, 48.4% had reduced HDL-C, 15.2% had overweight/central obesity, 2.3% had elevated TG, 10.0% had hyperglycemia, and 17.3% had hypertension. The median values of the three novel inflammatory metabolic parameters were 1.6 for LHR, 203.5 for PHR, and 141.4 for AISI (Table 1). During a median follow-up of 2.8 years, 116 PLWH (14.3%) developed MAFLD, corresponding to an incidence rate of 50.4 per 1,000 person-years. Compared with those without MAFLD, participants with MAFLD were more likely to have MSM as the transmission route, reduced HDL-C, and overweight/central obesity, and had higher levels of HGB, LYMPH, MONO, CD4 T cell counts, LHR, and PHR.

**Table 1:** Baseline characteristics of PLWH with and without MAFLD.

Variables	Overall (*n* = 814)	Non-MAFLD (*n* = 698)	MAFLD (*n* = 116)	*p* Value
Sex (n), %				0.308
Male	703 (86.4)	599 (85.8)	104 (89.7)	
Female	111 (13.6)	99 (14.2)	12 (10.3)	
Age (years), median (IQR)	37(28,49)	36 (28,49)	38 (28,47)	0.707
HIV transmission risk (n), %				0.031
Other/unknown	70 (8.6)	64 (9.2)	6 (5.2)	
MSM	380 (46.7)	313 (44.8)	67 (57.8)	
Heterosexual	364 (44.7)	321 (46.0)	43 (37.1)	
ART regimen (n), %				0.263
NNRTIs+NRTIs	541 (66.5)	467 (66.9)	74 (63.8)	
PIs + NRTIs	27 (3.3)	26 (3.7)	1 (0.9)	
3TC + DTG	37 (4.5)	32 (4.6)	5 (4.3)	
INSTIs+NRTIs	209 (25.7)	173 (24.8)	36 (31.0)	
Baseline HIV viral load (n), %				0.267
Viral suppression	476 (58.5)	403 (57.7)	73 (62.9)	
Low HIV viral load	112 (13.8)	94 (13.5)	18 (15.5)	
High HIV viral load	226 (27.8)	201 (28.8)	25 (21.6)	
Reduced HDL (n), %				<0.001
No	420 (51.6)	378 (54.2)	42 (36.2)	
Yes	394 (48.4)	320 (45.8)	74 (63.8)	
Overweight/central obesity (n), %				<0.001
No	690 (84.8)	610 (87.4)	80 (69.0)	
Yes	124 (15.2)	88 (12.6)	36 (31.0)	
Elevated TG (n), %				0.093
No	795 (97.7)	679 (97.3)	116 (100.0)	
Yes	19 (2.3)	19 (2.7)	0 (0.0)	
Hyperglycemia (n), %				0.856
No	733 (90.0)	628 (90.0)	105 (90.5)	
Yes	81 (10.0)	70 (10.0)	11 (9.5)	
Hypertension (n), %				0.353
No	673 (82.7)	573 (82.1)	100 (86.2)	
Yes	141 (17.3)	125 (17.9)	16 (13.8)	
HGB (g/L), median (IQR)	150(140,158)	150 (139,158)	153 (143,160)	0.004
PLT (10^9^/L), median (IQR)	219(184,257)	218 (181,257)	232(192,258)	0.066
NEUT (10^9^/L), median (IQR)	2.8(2.2,3.6)	2.8 (2.2,3.5)	2.9(2.3,3.8)	0.052
LYMPH (10^9^/L), median (IQR)	1.7(1.3,2.1)	1.6 (1.3,2.1)	2(1.5,2.5)	<0.001
MONO (10^9^/L), median (IQR)	0.39(0.31,0.48)	0.38 (0.31,0.48)	0.43(0.35,0.54)	<0.001
EO (10^9^/L), median (IQR)	0.08(0.05,0.15)	0.08 (0.05,0.15)	0.09(0.05,0.15)	0.678
BASO (10^9^/L), median (IQR)	0.02(0.02,0.04)	0.02 (0.02,0.04)	0.03(0.02,0.04)	0.209
CD4 T cell count (cells/mL), median (IQR)	365(237,489)	352 (233,476)	429(272,582)	0.002
LHR, median (IQR)	1.6(1.1,2.2)	1.5 (1.1,2.1)	2.1(1.5,2.8)	<0.001
PHR, median (IQR)	203.5(161.4,260.7)	200.0 (155.5,253.1)	221.2(188.7,284.2)	<0.001
AISI, median (IQR)	141.4(91.4,225.9)	139.8 (91.0,223.1)	153.5(96.3,235.9)	0.309

PLWH, people living with HIV; MAFLD, metabolic dysfunction-associated fatty liver disease; MSM, men who have sex with men; ART, antiretroviral therapy; NNRTIs, non-nucleoside reverse transcriptase inhibitors; NRTIs, nucleoside reverse transcriptase inhibitors; PIs, protease inhibitors; 3TC, lamivudine; DTG, dolutegravir; INSTIs, integrase strand transfer inhibitors; HDL-C, high-density lipoprotein cholesterol; TG, triglycerides; HGB, hemoglobin; PLT, platelet count; NEUT, neutrophil count; LYMPH, lymphocyte count; MONO, monocyte count; EO, eosinophil count; BASO, basophil count; LHR, lymphocyte-to-high-density lipoprotein cholesterol ratio; PHR, platelet to high-density lipoprotein cholesterol ratio; AISI, aggregate index of systemic inflammation.

Baseline characteristics stratified by tertiles of LHR, PHR, and AISI are shown in Supplementary Tables S1–S3. In Supplementary Table S1, higher LHR levels were significantly associated with male sex, younger age, MSM transmission route, 3TC + DTG and INSTIs+NRTIs regimens, reduced HDL-C, and elevated levels of HGB, PLT, NEUT, LYMPH, MONO, EO, BASO, and CD4 T cell counts. As shown in Supplementary Table S2, PHR displayed patterns similar to LHR, except that associations with HGB and ART regimen were not statistically significant. In Supplementary Table S3, higher AISI levels were significantly associated with older age, Heterosexual transmission route, lower lymphocyte levels, and higher levels of HGB, PLT, NEUT, MONO, EO, and BASO.

### 3.2 Risk factors analysis of MAFLD

**Figure 2:**
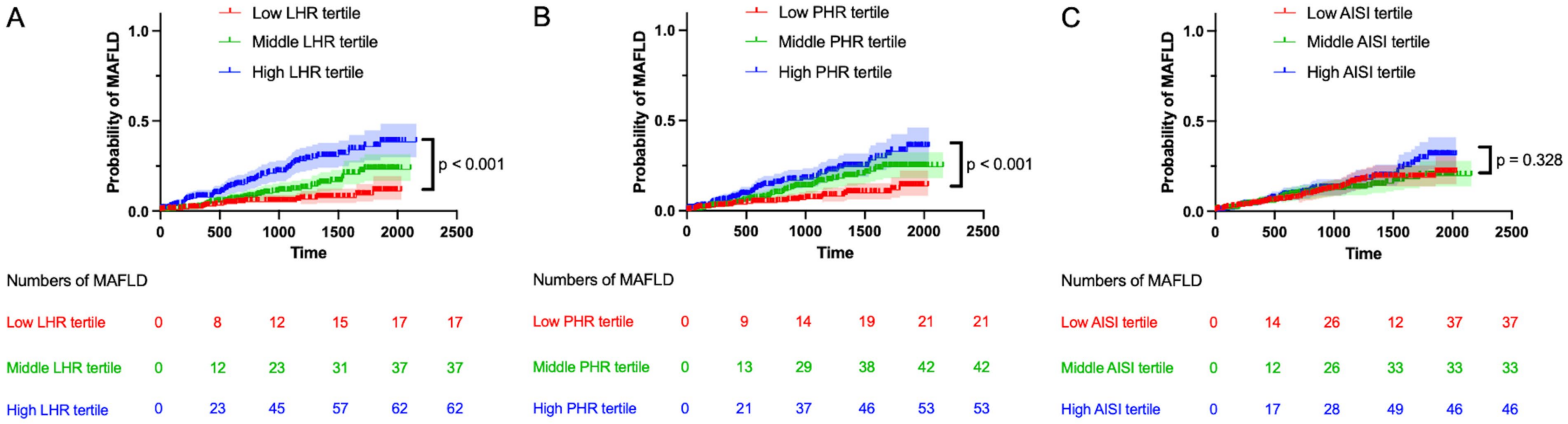
Kaplan–Meier curves show the probability of MAFLD in PLWH across tertiles of inflammatory metabolic parameters. **(A)** Probability of MAFLD by LHR tertiles: low (LHR < 1.25), middle (1.25 ≤ LHR < 1.94), high (LHR ≥ 1.94). **(B)** Probability of MAFLD by PHR tertiles: low (PHR < 178.26), middle (178.26 ≤ PHR < 234.40), high (PHR ≥ 234.40). **(C)** Probability of MAFLD by AISI tertiles: low (AISI <106.32), middle (106.32 ≤ AISI <187.74), high (AISI ≥187.74). LHR, lymphocyte-to-high-density lipoprotein cholesterol ratio; PHR, platelet to high-density lipoprotein cholesterol ratio; AISI, aggregate index of systemic inflammation.

As shown in Figure 2, Kaplan–Meier analysis revealed that the cumulative incidence of MAFLD increased significantly across tertiles of both LHR and PHR (log-rank test, *p* < 0.001 for both). PLWH in the highest tertiles of LHR and PHR consistently showed the highest incidence of MAFLD (Figures 2A,B). In contrast, no significant association was observed between AISI tertiles and MAFLD incidence (Figure 2C).

Univariate and multivariate Cox regression analyses were performed to validate the predictive value of inflammatory metabolic parameters for MAFLD (Table 2). Univariate Cox regression (Supplementary Figure S1 and Model 1) identified several risk factors for MAFLD, including 3TC + DTG and INSTIs+NRTIs regimens, reduced HDL-C, overweight/central obesity, HGB, NEUT, LYMPH, MONO, CD4 T cell count, and higher LHR and PHR tertiles. Specifically, compared with the lowest tertile, the middle and high LHR tertiles were associated with markedly increased risks of MAFLD (HR = 2.149, 95% CI: 1.210–3.816, *p* = 0.009; HR = 4.346, 95% CI: 2.541–7.434, *p* < 0.001), with similar associations observed for PHR (HR = 2.045, 95% CI: 1.211–3.452, *p* = 0.007 for middle vs. low; HR = 2.959, 95% CI: 1.785–4.907, *p* < 0.001 for high vs. low). After adjustment for age and sex in Model 2, these associations remained robust: middle and high LHR tertiles (HR = 2.292, 95% CI: 1.279–4.109, *p* = 0.005; HR = 4.730, 95% CI: 2.711–8.253, *p* < 0.001) and middle and high PHR tertiles (HR = 2.084, 95% CI: 1.228–3.535, *p* = 0.007; HR = 3.013, 95% CI: 1.804–5.033, *p* < 0.001) remained significantly associated with MAFLD. In the fully adjusted Model 3, the high LHR tertile remained an independent risk factor for MAFLD (HR = 2.315, 95% CI: 1.208–4.436, *p* = 0.011). For PHR, however, only the middle tertile (HR = 1.875, 95% CI: 1.083–3.245, *p* = 0.025) remained significant, whereas the association with the high tertile was attenuated. Across all three models, AISI showed no predictive value for MAFLD. VIF analysis confirmed the absence of significant multicollinearity between AISI and major metabolic or ART-related covariates (all VIF < 10), suggesting that the null association was unlikely due to overlapping predictor effects.

**Table 2:** Cox regression analysis of inflammatory metabolic parameters for MAFLD in PLWH.

Subgroup	Model 1		Model 2		Model 3	
	HR (95% CI)	*p* value	HR (95% CI)	*p* value	HR (95% CI)	*p* value
LHR						
Low tertile	Reference		Reference		Reference	
Middle tertile	2.149(1.210–3.816)	0.009	2.292(1.279–4.109)	0.005	1.609(0.873–2.965)	0.127
High tertile	4.346(2.541–7.434)	<0.001	4.730(2.711–8.253)	<0.001	2.315(1.208–4.436)	0.011
PHR						
Low tertile	Reference		Reference		Reference	
Middle tertile	2.045(1.211–3.452)	0.007	2.084(1.228–3.535)	0.007	1.875(1.083–3.245)	0.025
High tertile	2.959(1.785–4.907)	<0.001	3.013(1.804–5.033)	<0.001	1.727(0.945–3.157)	0.076
AISI						
Low tertile	Reference		Reference		Reference	
Middle tertile	0.918(0.574–1.467)	0.72	0.914(0.571–1.461)	0.706	0.843(0.523–1.361)	0.485
High tertile	1.263(0.819–1.947)	0.29	1.257(0.814–1.940)	0.303	1.162(0.747–1.807)	0.505

PLWH, people living with HIV; MAFLD, metabolic dysfunction-associated fatty liver disease. LHR, lymphocyte-to-high-density lipoprotein cholesterol ratio; PHR, platelet to high-density lipoprotein cholesterol ratio; AISI, aggregate index of systemic inflammation. Model 1 = unadjusted. Model 2 = adjusted for age and sex. Model 3 = adjusted for age, sex, ART regimen, reduced HDL-C, overweight/central obesity, elevated TG, hyperglycemia, hypertension, HGB, and CD4 T cell count. LHR tertiles: Low (<1.25), Middle (1.25–1.94), High (≥1.94). PHR tertiles: Low (<178.26), Middle (178.26–234.40), High (≥234.40). AISI tertiles: Low (<106.32), Middle (106.32–187.74), High (≥187.74).

### 3.3 RCS analysis of inflammatory metabolic parameters

**Figure 3:**
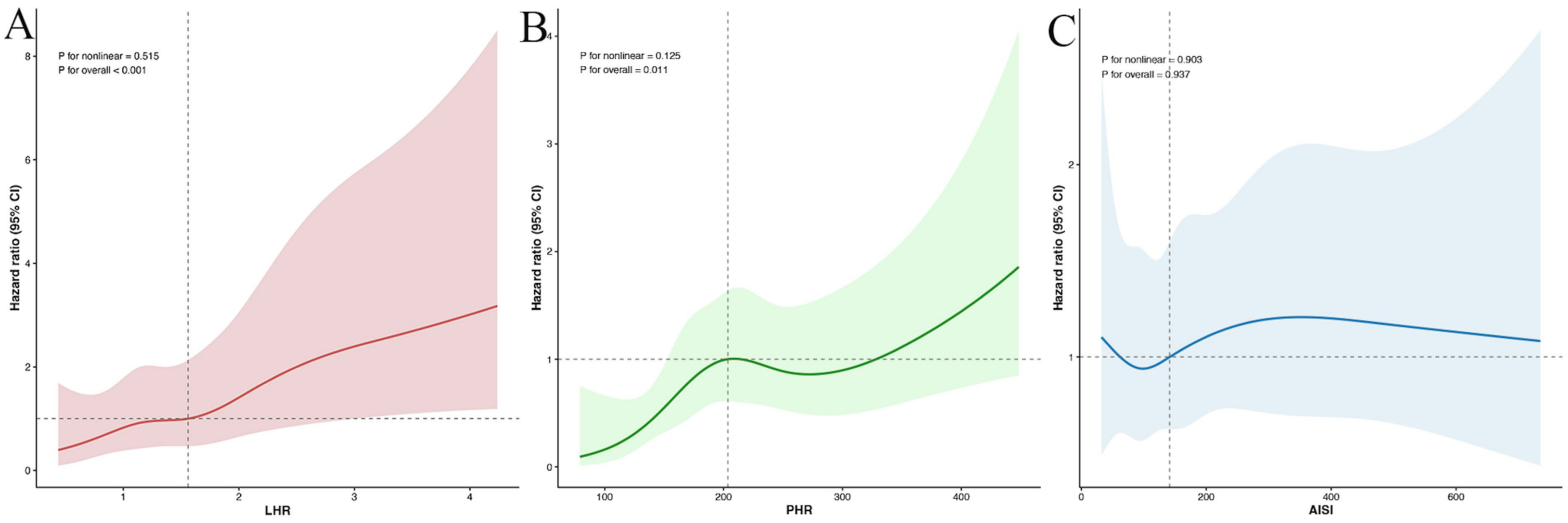
RCS analysis of the association between **(A)** lymphocyte-to-high-density lipoprotein cholesterol ratio (LHR), **(B)** platelet to high-density lipoprotein cholesterol ratio (PHR), and **(C)** aggregate index of systemic inflammation (AISI) and MAFLD in PLWH. Four knots were placed at the 5th, 35th, 65th, and 95th percentiles to generate smooth curves. The shaded areas represent 95% CI. Models were adjusted for age, sex, ART regimen, reduced HDL-C, overweight/central obesity, elevated TG, hyperglycemia, hypertension, HGB, and CD4 T cell count.

To further examine potential non-linear relationships, multivariable-adjusted RCS analyses were performed for LHR, PHR, and AISI in relation to incident MAFLD (Figure 3). Four knots were placed at the 5th, 35th, 65th, and 95th percentiles to generate smooth curves. No significant non-linear associations were observed between any of the three inflammatory metabolic parameters and MAFLD risk (all P for non-linearity >0.05). Consistent with Cox regression results, no linear or non-linear association was observed between AISI and MAFLD (P for overall association >0.05). These findings indicate that LHR and PHR showed linear associations with MAFLD risk, whereas AISI had no predictive value in this cohort.

### 3.4 ROC analysis of inflammatory metabolic parameters

**Figure 4:**
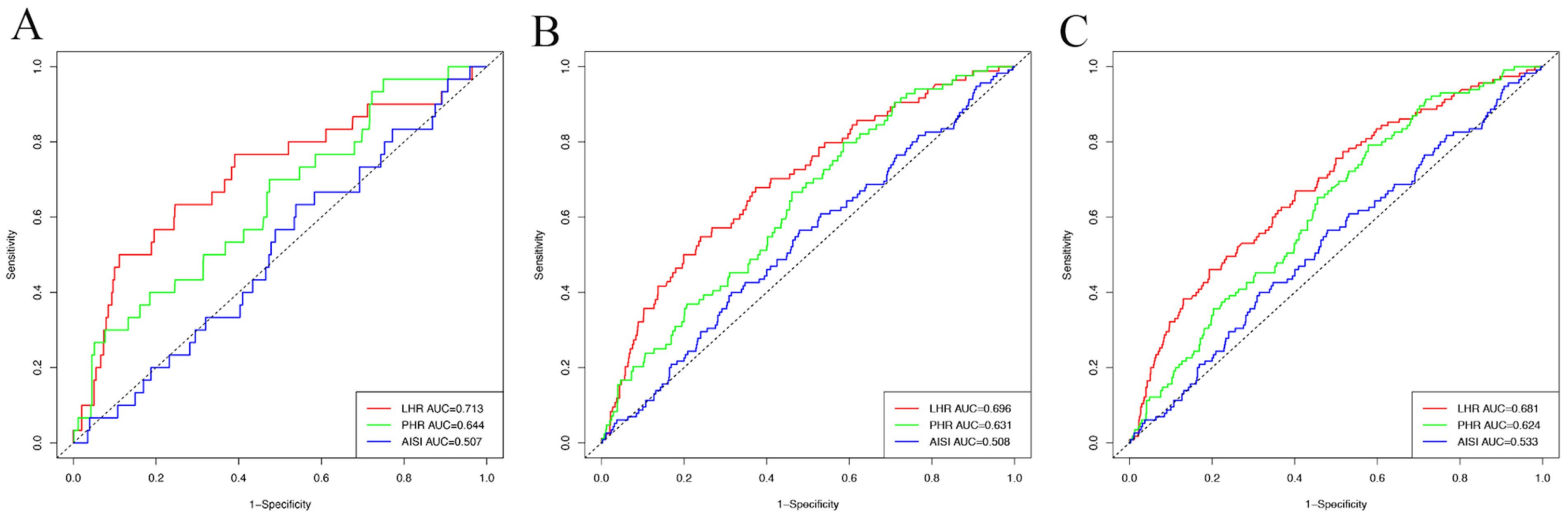
Time-dependent ROC curves evaluating the incidence of MAFLD at 1 year **(A)**, 3 years **(B)**, and 5 years **(C)** for the three inflammatory metabolic parameters. LHR, lymphocyte-to-high-density lipoprotein cholesterol ratio; PHR, platelet to high-density lipoprotein cholesterol ratio; AISI, aggregate index of systemic inflammation.

Time-dependent ROC analyses were performed to evaluate the predictive value of LHR, PHR, and AISI for incident MAFLD at 1, 3, and 5 years (Figure 4). Among the three parameters, LHR showed the strongest short-term predictive ability, with an area under the curve (AUC) of 0.713 (95% CI: 0.609–0.818) for 1-year incidence. PHR also showed moderate accuracy at 1 year (AUC = 0.644, 95% CI: 0.544–0.744). However, the predictive performance of both LHR and PHR declined over longer follow-up, suggesting greater utility for early risk stratification. In contrast, AISI consistently showed poor predictive value, with AUCs below 0.600 at 1, 3, and 5 years, indicating limited ability to discriminate MAFLD risk in this cohort.

### 3.5 Sensitivity analysis

To evaluate the robustness of our findings, subgroup analyses were conducted stratified by sex, age, HIV transmission risk, ART regimen, baseline HIV viral load, CD4 T cell count and metabolic comorbidities (Supplementary Figures S2–S4). Across all subgroups, LHR and PHR consistently remained significant risk factors for incident MAFLD, confirming the stability of these associations. In addition, the positive association between LHR and MAFLD remained robust across all CD4 strata, suggesting that its predictive effect was independent of baseline immune status. Except for reduced HDL-C, no significant interactions were observed between LHR and other covariates (all P for interaction >0.05). Similarly, no significant interactions were detected between PHR and any examined covariates, indicating that the associations of LHR and PHR with MAFLD were not materially influenced by baseline characteristics or comorbidities. In contrast, AISI showed no significant interactions with any covariates (all *p* > 0.05) and no association with MAFLD in any subgroup (all *p* > 0.05). These findings underscore the robustness of the associations between LHR and PHR and the risk of MAFLD.

## 4 Discussion

In this retrospective cohort study of 814 PLWH on ART, we evaluated the predictive value of three inflammatory metabolic parameters—LHR, PHR, and AISI—for the development of MAFLD. Among these parameters, LHR, which integrates immune function via lymphocyte with lipid metabolism reflected by HDL-C, showed the strongest predictive value. This association remained robust across subgroup analyses. PHR, which reflects the interplay between metabolic and coagulation status, also showed predictive potential, although its discriminative ability in time-dependent ROC analysis was weaker than LHR. In contrast, AISI, which reflects systemic inflammatory burden, showed no significant association with MAFLD in Cox regression or sensitivity analyses. These findings underscore the potential of LHR and PHR as accessible, cost-effective biomarkers for early identification of MAFLD risk in PLWH.

Recent studies have highlighted the close relationship of lymphocytes, platelets, and HDL-C with MAFLD progression (14–16), and composite parameters derived from these parameters have shown superior predictive ability. Tang et al. (10), using NHANES data, reported a significant positive association between LHR and MASLD (OR: 1.64, 95% CI: 1.40–1.92) in multivariable-adjusted logistic regression and identified an inverted L-shaped relationship between controlled attenuation parameter and LHR using a two-piece linear regression model. Moreover, LHR has also shown predictive potential for other metabolic diseases, including cardiovascular–kidney–metabolic syndrome (17), type 2 diabetes mellitus (18), and metabolic syndrome (19), underscoring its role as a marker of immune-metabolic dysregulation. Similarly, Lu et al. (11), also using NHANES data, found that elevated PHR was associated with increased prevalence of NAFLD and liver fibrosis, with RCS analysis revealing an S-shaped relationship between PHR and NAFLD risk, with a threshold of 181. Notably, although our study supports significant positive associations of LHR and PHR with MAFLD in PLWH, we found no evidence of non-linear relationships in RCS analyses. Differences in study populations may explain these discrepancies: our cohort consisted of PLWH with a median LHR of 1.6 (IQR: 1.1–2.2), much higher than the mean LHR of 0.46 ± 0.88 reported by Tang et al., whereas the median PHR in our study was 203.5, exceeding the threshold (>181) identified in Lu’s analysis as associated with higher NAFLD risk. Such differences in exposure ranges, together with distinct immunometabolic characteristics of PLWH, may account for these divergent findings. In contrast, previous studies have reported strong associations between AISI and MAFLD in the general population. For example, Zhang et al. (20) reported that higher AISI levels were independently associated with increased risk of fatty liver disease in a US cohort, even after adjusting for sex, age, BMI, hypertension, and diabetes. Similarly, a large study of 34,303 Chinese patients with hypertension found that each standard deviation increase in AISI, systemic inflammatory response index, and systemic immune-inflammation index was associated with 74, 62, and 58% higher odds of MAFLD, respectively, with AISI showing the strongest association (12). However, these studies did not directly compare AISI with metabolic-inflammatory markers such as LHR or PHR and were conducted in hypertensive or general populations. In our study, AISI levels were comparable between the MAFLD and non-MAFLD groups (median 153.5 [IQR 96.3–235.9] vs. 139.8 [IQR 91.0–223.1], *p* = 0.309), suggesting a relatively narrow spectrum of systemic inflammation among PLWH on long-term ART. This limited variability may reduce the discriminatory capacity of AISI in this population. Moreover, VIF analysis revealed no significant multicollinearity between AISI and baseline variables—including ART regimen, reduced HDL-C, overweight/central obesity, elevated TG, hyperglycemia, hypertension, HGB, and CD4 T cell count (all VIF < 10)—indicating that the null association was not attributable to collinearity. The attenuated association between AISI and MAFLD may also reflect the chronic immunologic alterations characteristic of PLWH. Despite effective ART and partial immune reconstitution, PLWH exhibit a persistent low-grade inflammatory state with limited fluctuation in systemic immune activation. This stable inflammatory milieu may reduce the sensitivity of general inflammatory indices such as AISI, which primarily capture temporal variations in systemic inflammation, thereby explaining their weaker predictive performance compared with inflammatory metabolic markers like LHR and PHR. Taken together, our findings suggest that LHR provides a more targeted and informative measure of immune-metabolic interactions in relation to MAFLD risk in PLWH.

The pathogenesis of MAFLD in PLWH is multifactorial, involving both inflammatory and metabolic pathways. Chronic HIV infection induces persistent immune activation and low-grade systemic inflammation, even under effective ART, contributing to hepatocellular injury and metabolic derangements (21). Systemic inflammation in PLWH is marked by elevated cytokines, including interleukin-6 and tumor necrosis factor-alpha (22). These proinflammatory mediators impair adipocyte function and stimulate lipolysis, increasing circulating free fatty acids that are deposited in the liver (23). In parallel, metabolic factors also play a pivotal role. Both HIV infection and long-term ART exposure are associated with key metabolic abnormalities—including hypertension, hyperglycemia, central obesity, and dyslipidemia—that are established risk factors for MAFLD (24). In our Cox regression analysis, ART regimens containing INSTIs, specifically 3TC + DTG and INSTIs+NRTIs, were significant risk factors for MAFLD. This finding is consistent with growing evidence that INSTIs are associated with substantial weight gain in PLWH (25, 26), and, more recently, with increased risk of MAFLD (27, 28). The mechanisms by which INSTIs contribute to MAFLD are multifactorial, including mitochondrial dysfunction, oxidative stress, adipokine imbalance, and ART-related weight gain, which collectively disrupt hepatic and systemic metabolic homeostasis (23). Taken together, these findings underscore the dual role of inflammatory and metabolic pathways in driving MAFLD pathogenesis in PLWH. A better understanding of these overlapping mechanisms is essential to guide the development of targeted interventions, including anti-inflammatory therapies, metabolic modulation strategies, and optimization of ART regimens.

Previous studies have shown that a higher baseline CD4 T cell count is associated with an increased risk of MAFLD and metabolic abnormalities in PLWH (29, 30). Consistent with these findings, our study observed higher CD4 T cell count and lymphocyte levels among individuals who developed MAFLD, possibly reflecting the combined effects of immune reconstitution and metabolic adaptation during ART. To further assess the potential confounding effect of immune status, we conducted a sensitivity analysis stratified by baseline CD4 T cell count. The positive association between LHR and MAFLD remained robust across all CD4 strata, indicating that this relationship was independent of immune reconstitution status. For PHR, a significant association with MAFLD was observed only among participants with a CD4 T cell count above 200 cells/μL, suggesting that baseline immune function may modify this relationship. Previous studies have also shown that a higher CD4 T cell count predicts greater immune recovery (31), and that INSTI use is associated with enhanced immune reconstitution (32). Both immune recovery (33) and INSTI exposure (27, 28) have been linked to adipose redistribution, dyslipidemia, and hepatic steatosis, supporting the notion that LHR and PHR reflect integrated immune–metabolic responses underlying hepatic steatosis in PLWH. Although LHR and PHR showed significant predictive value for incident MAFLD in PLWH (AUCs 0.713 and 0.644 at 1 year, respectively), their discriminatory performance declined over time. This indicates that although promising, these indices require further refinement to improve long-term predictive accuracy. The novel inflammatory metabolic parameters proposed in this study may aid early risk stratification for MAFLD in PLWH, particularly in resource-limited settings where advanced diagnostic tools are unavailable. Currently, risk prediction models specifically tailored to MAFLD in PLWH remain scarce. Most existing models were developed in the general population and rely primarily on conventional metabolic indicators such as body mass index, lipid profiles, and glucose levels (34–36). Within this context, LHR and PHR may offer complementary predictive value by capturing immune–metabolic interactions unique to PLWH. Their predictive accuracy could be improved by integrating these indices with established metabolic indicators and ART-related factors.

Clinically, our findings have important implications for improving early detection and personalized management of MAFLD in PLWH. As LHR and PHR can be easily obtained from routine, low-cost laboratory tests, they serve as practical and cost-effective tools for identifying individuals who may require closer clinical monitoring during ART. Integrating these biomarkers into routine follow-up protocols could facilitate early recognition of subclinical hepatic steatosis and metabolic deterioration, enabling clinicians to initiate lifestyle interventions, optimize ART regimens, and implement targeted metabolic strategies before irreversible liver injury occurs. Moreover, the use of LHR and PHR may enhance risk stratification and individualized follow-up, contributing to the advancement of precision medicine in managing metabolic complications among PLWH. Prospective studies are warranted to validate these findings and to explore their incorporation into standardized clinical algorithms for MAFLD risk assessment in this population.

This study has several limitations that warrant acknowledgment. First, as a single-center retrospective cohort conducted in a tertiary hospital, the generalizability of our findings to other populations should be interpreted with caution. Second, although we adjusted for multiple demographic, clinical, and metabolic confounders, residual or unmeasured confounding cannot be fully excluded. Although ART regimen was included in the multivariable models, detailed information on ART-related exposure—such as therapy duration, cumulative INSTIs exposure, use of tenofovir alafenamide, and longitudinal weight gain—was unavailable. These factors are well-established determinants of MAFLD and may have influenced the observed associations, potentially contributing to residual confounding. Third, the diagnosis of MAFLD in this study was based on abdominal ultrasonography, which, although practical for large-scale studies, has only moderate sensitivity for detecting mild hepatic steatosis and does not assess liver fibrosis. Consequently, misclassification bias cannot be excluded and may have affected both incidence estimates and the observed associations. Given the growing clinical relevance of fibrosis staging in MAFLD management, the lack of fibrosis assessment represents a limitation. Future research should validate our findings in larger, multicenter, prospective cohorts incorporating detailed ART exposure data, longitudinal metabolic monitoring, and advanced imaging or non-invasive biomarkers for both steatosis and fibrosis, to enhance generalizability and strengthen causal inference in PLWH.

## 5 Conclusion

In this retrospective cohort of PLWH on ART, LHR and PHR were independent predictors of incident MAFLD, with LHR showing the strongest and most consistent predictive value. AISI was not associated with MAFLD. These findings highlight the importance of assessing inflammatory metabolic markers in PLWH and suggest that LHR and PHR, readily obtainable from routine low-cost laboratory tests, may serve as practical tools for identifying individuals who could benefit from closer monitoring and early intervention.

## Data Availability

The raw data supporting the conclusions of this article will be made available by the authors, without undue reservation.

## References

[1] Verna EC. Non-alcoholic fatty liver disease and non-alcoholic steatohepatitis in patients with HIV. Lancet Gastroenterol Hepatol. (2017) 2:211–23. doi: 10.1016/S2468-1253(16)30120-0, 28404136

[2] Eslam M Sanyal AJ George J. MAFLD: a consensus-driven proposed nomenclature for metabolic associated fatty liver disease. Gastroenterology. (2020) 158:1999–2014.e1. doi: 10.1053/j.gastro.2019.11.312, 32044314

[3] Cervo A Milic J Mazzola G Schepis F Petta S Krahn T . Prevalence, predictors, and severity of lean nonalcoholic fatty liver disease in patients living with human immunodeficiency virus. Clin Infect Dis. (2020) 71:e694–701. doi: 10.1093/cid/ciaa430, 32280969

[4] Younossi ZM. Non-alcoholic fatty liver disease - a global public health perspective. J Hepatol. (2019) 70:531–44. doi: 10.1016/j.jhep.2018.10.033, 30414863

[5] Li J Zhou J Li P Wang Y Ridderhof N Al-Tawfiq JA . The global prevalence and impact of steatotic liver disease and viral infections: a systematic review and meta-analysis. Hepatol Commun. (2025) 9:e0689. doi: 10.1097/HC9.0000000000000689 40227096 PMC11999411

[6] Navarro J. HIV and liver disease. AIDS Rev. (2022) 25:87–96. doi: 10.24875/AIDSRev.M22000052, 35901073

[7] Olveira A Augustin S Benlloch S Ampuero J Suárez-Pérez JA Armesto S . The essential role of IL-17 as the pathogenetic link between psoriasis and metabolic-associated fatty liver disease. Life Basel. (2023) 13:419. doi: 10.3390/life13020419, 36836776 PMC9963792

[8] Biały M Czarnecki M Inglot M. Impact of combination antiretroviral treatment on liver metabolic health in HIV-infected persons. Viruses. (2023) 15:2432. doi: 10.3390/v15122432, 38140673 PMC10747352

[9] Agarwal N Iyer D Patel SG Sekhar RV Phillips TM Schubert U . HIV-1 Vpr induces adipose dysfunction in vivo through reciprocal effects on PPAR/GR co-regulation. Sci Transl Med. (2013) 5:213ra164. doi: 10.1126/scitranslmed.3007148, 24285483 PMC4009012

[10] Tang C Peng D Zong K Wu Z Gong M Li H . Association between the lymphocyte-to-high-density lipoprotein ratio and metabolic dysfunction-associated steatotic liver disease among US adults: a cross-sectional study from NHANES 2017 to 2020. BMC Gastroenterol. (2024) 24:470. doi: 10.1186/s12876-024-03565-5, 39716074 PMC11667913

[11] Lu CF Cang XM Liu WS Wang LH Huang HY Sang SM . Association between the platelet/high-density lipoprotein cholesterol ratio and nonalcoholic fatty liver disease: results from NHANES 2017-2020. Lipids Health Dis. (2023) 22:130. doi: 10.1186/s12944-023-01861-9, 37568178 PMC10422828

[12] Shen D Cai X Hu J Song S Zhu Q Ma H . Inflammatory indices and MAFLD prevalence in hypertensive patients: a large-scale cross-sectional analysis from China. J Inflamm Res. (2025) 18:1623–38. doi: 10.2147/JIR.S503648 39925928 PMC11806676

[13] Chinese Society of hepatology CMA. Guidelines for the prevention and treatment of metabolic dysfunction-associated (non-alcoholic) fatty liver disease (version 2024). Zhonghua Gan Zang Bing Za Zhi. (2024) 32:418–34. doi: 10.3760/cma.j.cn501113-20240327-00163, 38858192 PMC12677420

[14] Pan H Liu B Luo X Shen X Sun J Zhang A. Non-alcoholic fatty liver disease risk prediction model and health management strategies for older Chinese adults: a cross-sectional study. Lipids Health Dis. (2023) 22:205. doi: 10.1186/s12944-023-01966-1, 38007441 PMC10675849

[15] Ramadori P Klag T Malek NP Heikenwalder M. Platelets in chronic liver disease, from bench to bedside. JHEP Rep. (2019) 1:448–59. doi: 10.1016/j.jhepr.2019.10.001, 32039397 PMC7005648

[16] Xuan Y Zhu M Xu L Huangfu S Li T Liu C . Elevated non-HDL-C to HDL-C ratio as a marker for NAFLD and liver fibrosis risk: a cross-sectional analysis. Front Endocrinol (Lausanne). (2024) 15:1457589. doi: 10.3389/fendo.2024.1457589, 39473504 PMC11518740

[17] Song J Xu Z Yu H Li A Liu Y Jin M. Association of three composite inflammatory and lipid metabolism indicators with cardiovascular-kidney-metabolic syndrome: a cross-sectional study based on NHANES 1999-2020. Mediat Inflamm. (2025) 2025:6691516. doi: 10.1155/mi/6691516 PMC12081155 40376312

[18] Li Y Guo X Ge J Li Q Chen X Zhu Y . Sex differences in associations of metabolic inflammation and insulin resistance with incident type 2 diabetes mellitus: a retrospective cohort of adults with annual health examinations. Lipids Health Dis. (2025) 24:50. doi: 10.1186/s12944-025-02473-1, 39953587 PMC11829553

[19] Guo J Mutailipu K Wen X Yin J You H Qu S . Association between lymphocyte to high-density lipoprotein cholesterol ratio and insulin resistance and metabolic syndrome in US adults: results from NHANES 2007-2018. Lipids Health Dis. (2025) 24:9. doi: 10.1186/s12944-024-02411-7, 39794792 PMC11721163

[20] Zhang M Yuan Y Wang C Huang Y Fan M Li X . Aggregate index of systemic inflammation tied to increased fatty liver disease risk: insights from NHANES data. BMC Gastroenterol. (2025) 25:399. doi: 10.1186/s12876-025-03998-6, 40410700 PMC12101034

[21] Bourgi K Wanjalla C Koethe JR. Inflammation and metabolic complications in HIV. Curr HIV/AIDS Rep. (2018) 15:371–81. doi: 10.1007/s11904-018-0411-2, 30058057

[22] Lu J Ma SS Zhang WY Duan JP. Changes in peripheral blood inflammatory factors (TNF-α and IL-6) and intestinal flora in AIDS and HIV-positive individuals. J Zhejiang Univ Sci B. (2019) 20:793–802. doi: 10.1631/jzus.B1900075, 31489799 PMC6751483

[23] Iacob SA Iacob DG. Non-alcoholic fatty liver disease in HIV/HBV patients - a metabolic imbalance aggravated by antiretroviral therapy and perpetuated by the hepatokine/adipokine axis breakdown. Front Endocrinol (Lausanne). (2022) 13:814209. doi: 10.3389/fendo.2022.814209, 35355551 PMC8959898

[24] Jin Y Zhu J Chen Q Wang M Shen Z Dong Y . Development and validation of a nomogram for predicting the outcome of metabolic syndrome among people living with HIV after antiretroviral therapy in China. Front Cell Infect Microbiol. (2025) 15:1514823. doi: 10.3389/fcimb.2025.1514823, 40051708 PMC11882517

[25] Eckard AR McComsey GA. Weight gain and integrase inhibitors. Curr Opin Infect Dis. (2020) 33:10–9. doi: 10.1097/qco.0000000000000616, 31789693 PMC7433018

[26] Jemal M. A review of dolutegravir-associated weight gain and secondary metabolic comorbidities. SAGE Open Med. (2024) 12:20503121241260613. doi: 10.1177/20503121241260613, 38881592 PMC11179510

[27] Kirkegaard-Klitbo DM Thomsen MT Gelpi M Bendtsen F Nielsen SD Benfield T. Hepatic steatosis associated with exposure to elvitegravir and raltegravir. Clin Infect Dis. (2021) 73:e811–4. doi: 10.1093/cid/ciab057, 33493297

[28] Bischoff J Gu W Schwarze-Zander C Boesecke C Wasmuth JC van Bremen K . Stratifying the risk of NAFLD in patients with HIV under combination antiretroviral therapy (cART). EClinMed. (2021) 40:101116. doi: 10.1016/j.eclinm.2021.101116, 34522873 PMC8427211

[29] Maurice JB Patel A Scott AJ Patel K Thursz M Lemoine M. Prevalence and risk factors of nonalcoholic fatty liver disease in HIV-monoinfection. AIDS. (2017) 31:1621–32. doi: 10.1097/QAD.0000000000001504, 28398960

[30] van Eekeren LE Vadaq N Vos W Blaauw MJT Groenendijk AL van Lunzen J . Liver steatosis is prevalent in lean people with HIV and associated with exposure to antiretroviral treatment-a cross-sectional study. Open Forum Infect Dis. (2024) 11:ofae266. doi: 10.1093/ofid/ofae266 38868310 PMC11167668

[31] Kouamou V Gundidza P Ndhlovu CE Makadzange AT. Effects of gender and baseline CD4 count on post-treatment CD4 count recovery and outcomes in patients with advanced HIV disease: a retrospective cohort study. AIDS Res Hum Retrovir. (2023) 39:340–9. doi: 10.1089/AID.2022.0117 36924288

[32] Han WM Avihingsanon A Rajasuriar R Tanuma J Mundhe S Lee MP . CD4/CD8 ratio recovery among people living with HIV starting with first-line integrase Strand transfer inhibitors: a prospective regional cohort analysis. J Acquir Immune Defic Syndr. (2023) 92:180–8. doi: 10.1097/QAI.0000000000003121, 36625858 PMC10064076

[33] Yan M Man S Ma L Guo L Huang L Gao W. Immunological mechanisms in steatotic liver diseases: an overview and clinical perspectives. Clin Mol Hepatol. (2024) 30:620–48. doi: 10.3350/cmh.2024.0315, 38988278 PMC11540396

[34] Lv T Tian J Sun Y Zhang Y Qi F Xiang L . Institutional nomogram for estimating risk of metabolic associated fatty liver disease (MAFLD). Diabetes Metab Syndr Obes. (2024) 17:3735–52. doi: 10.2147/DMSO.S469677 39403553 PMC11472740

[35] Yuan Y Xu M Zhang X Tang X Zhang Y Yang X . Development and validation of a nomogram model for predicting the risk of MAFLD in the young population. Sci Rep. (2024) 14:9376. doi: 10.1038/s41598-024-60100-y, 38654043 PMC11039663

[36] Yang M Chen X Shen Q Xiong Z Liu T Leng Y . Development and validation of a predictive nomogram for the risk of MAFLD in postmenopausal women. Front Endocrinol (Lausanne). (2024) 15:1334924. doi: 10.3389/fendo.2024.1334924, 39165508 PMC11334217

