# Supplementary material for: Novel inflammatory metabolic parameters as predictors of metabolic dysfunction-associated fatty liver disease in people living with HIV receiving antiretroviral therapy: a retrospective cohort study
Source: Front Public Health. 2026 Jan 21;14:1716876. doi: 10.3389/fpubh.2026.1716876 (PMC12868174; doi:10.3389/fpubh.2026.1716876)
Supplement: Supplementary file 1 [file Supplementary_file_1.docx]

**Table S1.** Baseline characteristics of PLWH stratified by LHR tertiles

|  | **Low LHR tertile** | **Middle LHR tertile** | **High LHR tertile** | **P Value** |
| --- | --- | --- | --- | --- |
| Sex (n), % |  |  |  | <0.001 |
| Male | 202 (74.3) | 243 (89.7) | 258 (95.2) |  |
| Female | 70 (25.7) | 28 (10.3) | 13 (4.8) |  |
| Age (years), median (IQR) | 42 (31,53) | 34 (27,46) | 33 (26,45) | <0.001 |
| HIV transmission risk (n), % |  |  |  | <0.001 |
| Other/unknown | 21 (7.7) | 25 (9.2) | 24 (8.9) |  |
| MSM | 91 (33.5) | 138 (50.9) | 151 (55.7) |  |
| Heterosexual | 160 (58.8) | 108 (39.9) | 96 (35.4) |  |
| ART regimen (n), % |  |  |  | 0.018 |
| NNRTIs+NRTIs | 178 (65.4) | 199 (73.4) | 164 (60.5) |  |
| PIs+NRTIs | 13 (4.8) | 6 (2.2) | 8 (3.0) |  |
| 3TC+DTG | 8 (2.9) | 11 (4.1) | 18 (6.6) |  |
| INSTIs+NRTIs | 73 (26.8) | 55 (20.3) | 81 (29.9) |  |
| Baseline HIV viral load (n), % |  |  |  | 0.051 |
| ﻿Viral suppression | 163 (59.9) | 162 (59.8) | 151 (55.7) |  |
| Low HIV viral load | 25 (9.2) | 39 (14.4) | 48 (17.7) |  |
| High HIV viral load | 84 (30.9) | 70 (25.8) | 72 (26.6) |  |
| Reduced HDL (n), % |  |  |  | <0.001 |
| No | 198 (72.8) | 150 (55.4) | 72 (26.6) |  |
| Yes | 74 (27.2) | 121 (44.6) | 199 (73.4) |  |
| Overweight/central obesity (n), % |  |  |  | 0.566 |
| No | 235 (86.4) | 230 (84.9) | 225 (83.0) |  |
| Yes | 37 (13.6) | 41 (15.1) | 46 (17.0) |  |
| Elevated TG (n), % |  |  |  | 0.503 |
| No | 268 (98.5) | 264 (97.4) | 263 (97.0) |  |
| Yes | 4 (1.5) | 7 (2.6) | 8 (3.0) |  |
| Hyperglycemia (n), % |  |  |  | 0.662 |
| No | 241 (88.6) | 246 (90.8) | 246 (90.8) |  |
| Yes | 31 (11.4) | 25 (9.2) | 25 (9.2) |  |
| Hypertension (n), % |  |  |  | 0.876 |
| No | 222 (81.6) | 226 (83.4) | 225 (83.0) |  |
| Yes | 50 (18.4) | 45 (16.6) | 46 (17.0) |  |
| HGB (g/L), median (IQR) | 145 (133,156) | 151 (141,158) | 153 (145,160) | <0.001 |
| PLT (10^9^/L), median (IQR) | 207 (173,248) | 218 (184,254) | 234 (200,264) | <0.001 |
| NEUT (10^9^/L), median (IQR) | 2.6 (2.1,3.4) | 2.9 (2.2,3.6) | 2.9 (2.4,3.6) | 0.002 |
| LYMPH (10^9^/L), median (IQR) | 1.1 (0.9,1.4) | 1.7 (1.5,1.9) | 2.3 (2.0,2.7) | <0.001 |
| MONO (10^9^/L), median (IQR) | 0.33 (0.28,0.41) | 0.40 (0.32,0.47) | 0.46 (0.37,0.55) | <0.001 |
| EO (10^9^/L), median (IQR) | 0.06 (0.03,0.13) | 0.08 (0.05,0.15) | 0.11 (0.06,0.18) | <0.001 |
| BASO (10^9^/L), median (IQR) | 0.02 (0.01,0.03) | 0.03 (0.02,0.03) | 0.03 (0.02,0.04) | <0.001 |
| CD4 T cell count (cells/mL), median (IQR) | 261 (162,387) | 384 (274,496) | 444 (310,584) | <0.001 |
| MAFLD rate, (n), % |  |  |  | <0.001 |
| No | 255 (93.8) | 234 (86.3) | 209 (77.1) |  |
| Yes | 17 (6.3) | 37 (13.7) | 62 (22.9) |  |

**Abbreviations**: PLWH, people living with HIV; LHR, lymphocyte-to-high-density lipoprotein cholesterol ratio; MSM, men who have sex with men; ART, antiretroviral therapy; NNRTIs, non-nucleoside reverse transcriptase inhibitors; NRTIs, nucleoside reverse transcriptase inhibitors; PIs, protease inhibitors; 3TC, lamivudine; DTG, dolutegravir; INSTIs, integrase strand transfer inhibitors; HDL-C, high-density lipoprotein cholesterol; TG, triglycerides; HGB, hemoglobin; PLT, platelet count; NEUT, neutrophil count; LYMPH, lymphocyte count; MONO, monocyte count; EO, eosinophil count; BASO, basophil count; MAFLD, metabolic dysfunction-associated fatty liver disease.

**Notes**: LHR tertiles: Low (<1.25), Middle (1.25–1.94), High (≥1.94).

**Table S2.** Baseline characteristics of PLWH stratified by PHR tertiles

|  | **Low PHR tertile** | **Middle PHR tertile** | **High PHR tertile** | **P Value** |
| --- | --- | --- | --- | --- |
| Sex (n), % |  |  |  | <0.001 |
| Male | 217 (79.8) | 233 (86.0) | 253 (93.4) |  |
| Female | 55 (20.2) | 38 (14.0) | 18 (6.6) |  |
| Age (years), median (IQR) | 41 (30,54) | 34 (27,47) | 34 (26,45) | <0.001 |
| HIV transmission risk (n), % |  |  |  | 0.015 |
| Other/unknown | 19 (7.0) | 26 (9.6) | 25 (9.2) |  |
| MSM | 108 (39.7) | 135 (49.8) | 137 (50.6) |  |
| Heterosexual | 145 (53.3) | 110 (40.6) | 109 (40.2) |  |
| ART regimen (n), % |  |  |  | 0.326 |
| NNRTIs+NRTIs | 176 (64.7) | 191 (70.5) | 174 (64.2) |  |
| PIs+NRTIs | 13 (4.8) | 6 (2.2) | 8 (3.0) |  |
| 3TC+DTG | 9 (3.3) | 12 (4.4) | 16 (5.9) |  |
| INSTIs+NRTIs | 74 (27.2) | 62 (22.9) | 73 (26.9) |  |
| Baseline HIV viral load (n), % |  |  |  | 0.146 |
| ﻿Viral suppression | 167 (61.4) | 166 (61.3) | 143 (52.8) |  |
| Low HIV viral load | 31 (11.4) | 34 (12.5) | 47 (17.3) |  |
| High HIV viral load | 74 (27.2) | 71 (26.2) | 81 (29.9) |  |
| Reduced HDL (n), % |  |  |  | <0.001 |
| No | 205 (75.4) | 163 (60.1) | 52 (19.2) |  |
| Yes | 67 (24.6) | 108 (39.9) | 219 (80.8) |  |
| Overweight/central obesity (n), % |  |  |  | 0.779 |
| No | 230 (84.6) | 233 (86.0) | 227 (83.8) |  |
| Yes | 42 (15.4) | 38 (14.0) | 44 (16.2) |  |
| Elevated TG (n), % |  |  |  | 0.499 |
| No | 268 (98.5) | 264 (97.4) | 263 (97.0) |  |
| Yes | 4 (1.5) | 7 (2.6) | 8 (3.0) |  |
| Hyperglycemia (n), % |  |  |  | 0.469 |
| No | 242 (89.0) | 249 (91.9) | 242 (89.3) |  |
| Yes | 30 (11.0) | 22 (8.1) | 29 (10.7) |  |
| Hypertension (n), % |  |  |  | 0.522 |
| No | 219 (80.5) | 228 (84.1) | 226 (83.4) |  |
| Yes | 53 (19.5) | 43 (15.9) | 45 (16.6) |  |
| HGB (g/L), median (IQR) | 149 (136,158) | 151 (142,159) | 150 (140,158) | 0.093 |
| PLT (10^9^/L), median (IQR) | 178 (148,208) | 221 (196,246) | 259 (230,295) | <0.001 |
| NEUT (10^9^/L), median (IQR) | 2.6 (2.0,3.3) | 2.8 (2.3,3.6) | 3.1 (2.4,3.9) | <0.001 |
| LYMPH (10^9^/L), median (IQR) | 1.5 (1.1,1.8) | 1.7 (1.3,2.1) | 1.9 (1.5,2.3) | <0.001 |
| MONO (10^9^/L), median (IQR) | 0.34 (0.28,0.43) | 0.39 (0.32,0.48) | 0.44 (0.36,0.54) | <0.001 |
| EO (10^9^/L), median (IQR) | 0.07 (0.03,0.13) | 0.08 (0.05,0.16) | 0.10 (0.06,0.17) | <0.001 |
| BASO (10^9^/L), median (IQR) | 0.02 (0.01,0.03) | 0.02 (0.02,0.04) | 0.03 (0.02,0.04) | <0.001 |
| CD4 T cell count (cells/mL), median (IQR) | 328 (212,441) | 368 (259,497) | 389 (242,531) | <0.001 |
| MAFLD rate, (n), % |  |  |  | <0.001 |
| No | 251 (92.3) | 229 (84.5) | 218 (80.4) |  |
| Yes | 21 (7.7) | 42 (15.5) | 53 (19.6) |  |

**Abbreviations**: PLWH, people living with HIV; PHR, platelet to high-density lipoprotein cholesterol ratio; MSM, men who have sex with men; ART, antiretroviral therapy; NNRTIs, non-nucleoside reverse transcriptase inhibitors; NRTIs, nucleoside reverse transcriptase inhibitors; PIs, protease inhibitors; 3TC, lamivudine; DTG, dolutegravir; INSTIs, integrase strand transfer inhibitors; HDL-C, high-density lipoprotein cholesterol; TG, triglycerides; HGB, hemoglobin; PLT, platelet count; NEUT, neutrophil count; LYMPH, lymphocyte count; MONO, monocyte count; EO, eosinophil count; BASO, basophil count; MAFLD, metabolic dysfunction-associated fatty liver disease.

**Notes**: PHR tertiles: Low (<178.26), Middle (178.26–234.40), High (≥234.40).

**Table S3.** Baseline characteristics of PLWH stratified by AISI tertiles

|  | **Low AISI tertile** | **Middle** **AISI tertile** | **High AISI tertile** | **P Value** |
| --- | --- | --- | --- | --- |
| Sex (n), % |  |  |  | 0.215 |
| Male | 227 (83.5) | 240 (88.6) | 236 (87.1) |  |
| Female | 45 (16.5) | 31 (11.4) | 35 (12.9) |  |
| Age (years), median (IQR) | 37 (28,51) | 33 (27,45) | 39 (28,51) | 0.007 |
| HIV transmission risk (n), % |  |  |  | 0.001 |
| Other/unknown | 16 (5.9) | 34 (12.5) | 20 (7.4) |  |
| MSM | 124 (45.6) | 140 (51.7) | 116 (42.8) |  |
| Heterosexual | 132 (48.5) | 97 (35.8) | 135 (49.8) |  |
| ART regimen (n), % |  |  |  | 0.722 |
| NNRTIs+NRTIs | 175 (64.3) | 181 (66.8) | 185 (68.3) |  |
| PIs+NRTIs | 12 (4.4) | 9 (3.3) | 6 (2.2) |  |
| 3TC+DTG | 11 (4.0) | 11 (4.1) | 15 (5.5) |  |
| INSTIs+NRTIs | 74 (27.2) | 70 (25.8) | 65 (24.0) |  |
| Baseline HIV viral load (n), % |  |  |  | 0.597 |
| ﻿Viral suppression | 160 (58.8) | 161 (59.4) | 155 (57.2) |  |
| Low HIV viral load | 40 (14.7) | 40 (14.8) | 32 (11.8) |  |
| High HIV viral load | 72 (26.5) | 70 (25.8) | 84 (31.0) |  |
| Reduced HDL (n), % |  |  |  | 0.874 |
| No | 140 (51.5) | 143 (52.8) | 137 (50.6) |  |
| Yes | 132 (48.5) | 128 (47.2) | 134 (49.4) |  |
| Overweight/central obesity (n), % |  |  |  | 0.481 |
| No | 225 (82.7) | 234 (86.3) | 231 (85.2) |  |
| Yes | 47 (17.3) | 37 (13.7) | 40 (14.8) |  |
| Elevated TG (n), % |  |  |  | 0.960 |
| No | 266 (97.8) | 265 (97.8) | 264 (97.4) |  |
| Yes | 6 (2.2) | 6 (2.2) | 7 (2.6) |  |
| Hyperglycemia (n), % |  |  |  | 0.326 |
| No | 249 (91.5) | 246 (90.8) | 238 (87.8) |  |
| Yes | 23 (8.5) | 25 (9.2) | 33 (12.2) |  |
| Hypertension (n), % |  |  |  | 0.075 |
| No | 225 (82.7) | 234 (86.3) | 214 (79.0) |  |
| Yes | 47 (17.3) | 37 (13.7) | 57 (21.0) |  |
| HGB (g/L), median (IQR) | 149 (138,156) | 152 (142,160) | 149 (139,158) | 0.003 |
| PLT (10^9^/L), median (IQR) | 191 (153,218) | 222 (190,257) | 249 (218,281) | <0.001 |
| NEUT (10^9^/L), median (IQR) | 2.1 (1.7,2.5) | 2.9 (2.5,3.3) | 3.8 (3.2,4.5) | <0.001 |
| LYMPH (10^9^/L), median (IQR) | 1.7 (1.4,2.2) | 1.8 (1.3,2.2) | 1.6 (1.2,2.0) | <0.001 |
| MONO (10^9^/L), median (IQR) | 0.32 (0.26,0.37) | 0.39 (0.33,0.47) | 0.48 (0.4,0.58) | <0.001 |
| EO (10^9^/L), median (IQR) | 0.08 (0.04,0.13) | 0.09 (0.05,0.16) | 0.08 (0.05,0.16) | 0.047 |
| BASO (10^9^/L), median (IQR) | 0.02 (0.01,0.03) | 0.03 (0.02,0.04) | 0.03 (0.02,0.04) | <0.001 |
| CD4 T cell count (cells/mL), median (IQR) | 350 (242,480) | 383 (249,522) | 345 (221,480) | 0.184 |
| MAFLD rate, (n), % |  |  |  | 0.267 |
| No | 235 (86.4) | 238 (87.8) | 225 (83.0) |  |
| Yes | 37 (13.6) | 33 (12.2) | 46 (17.0) |  |

**Abbreviations**: PLWH, people living with HIV; AISI, aggregate index of systemic inflammation; MSM, men who have sex with men; ART, antiretroviral therapy; NNRTIs, non-nucleoside reverse transcriptase inhibitors; NRTIs, nucleoside reverse transcriptase inhibitors; PIs, protease inhibitors; 3TC, lamivudine; DTG, dolutegravir; INSTIs, integrase strand transfer inhibitors; HDL-C, high-density lipoprotein cholesterol; TG, triglycerides; HGB, hemoglobin; PLT, platelet count; NEUT, neutrophil count; LYMPH, lymphocyte count; MONO, monocyte count; EO, eosinophil count; BASO, basophil count; MAFLD, metabolic dysfunction-associated fatty liver disease.

**Notes**: AISI tertiles: Low (<106.32), Middle (106.32–187.74), High (≥187.74).


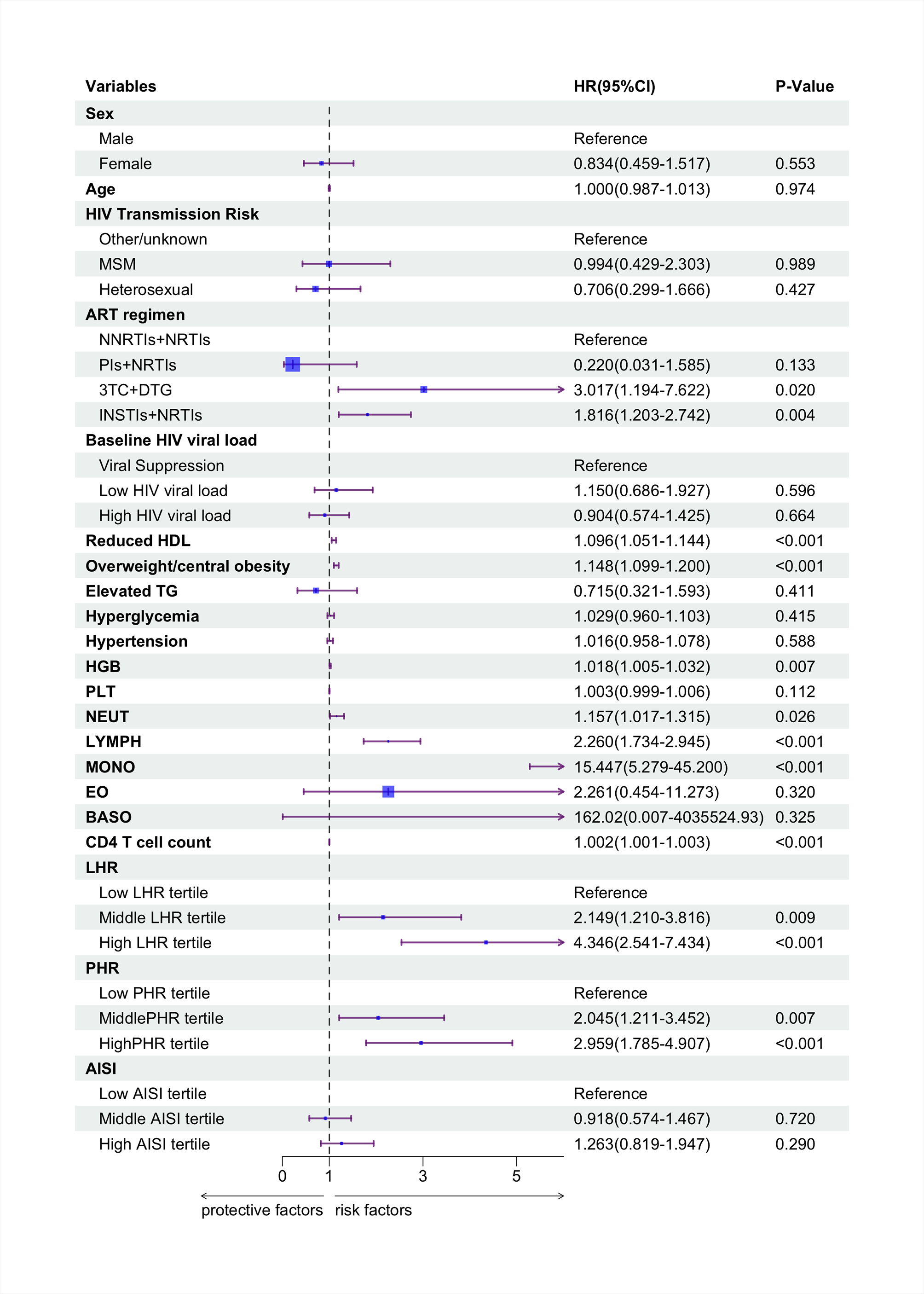
**Figure S1.** Univariate Cox regression analysis of MAFLD in PLWH.


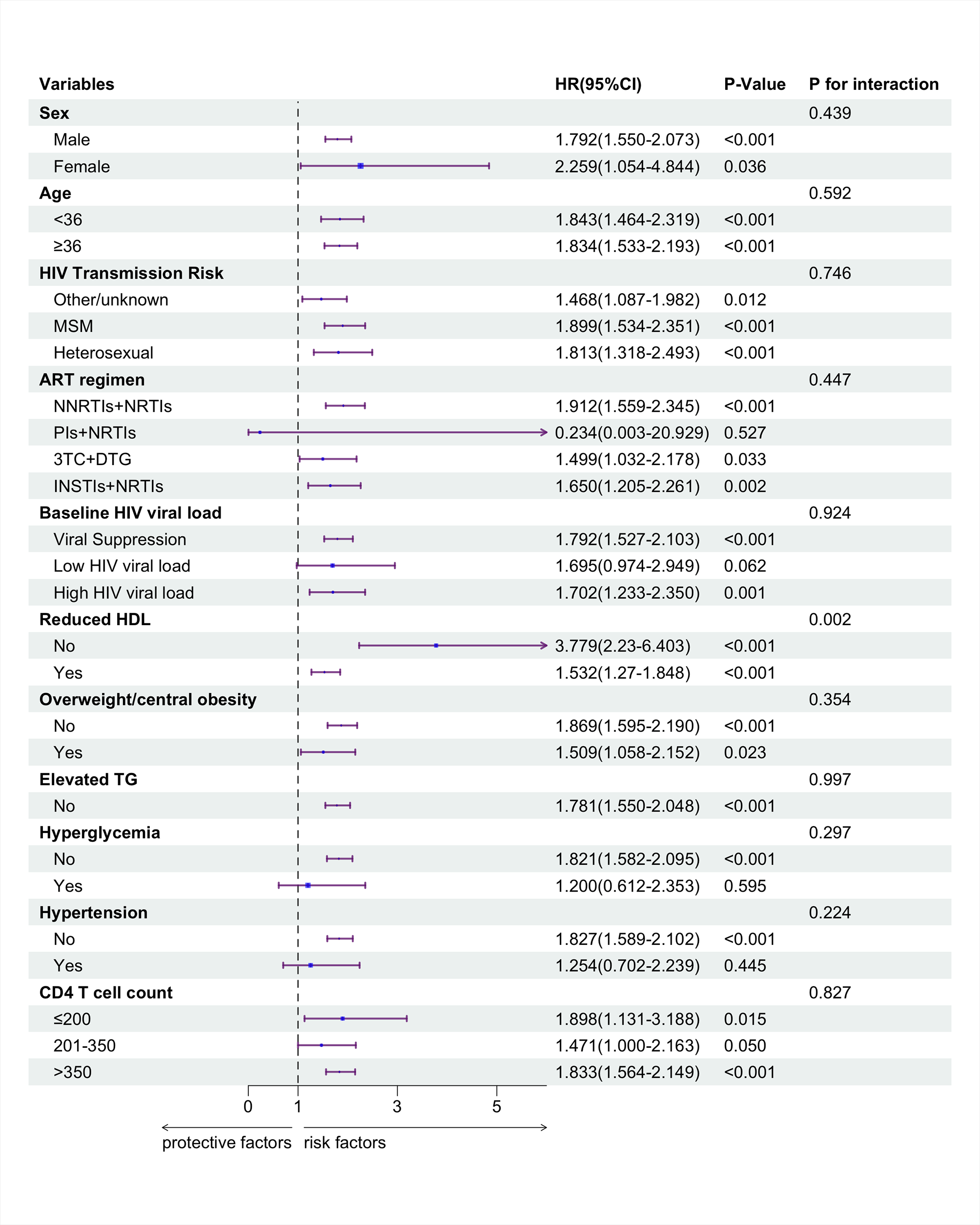
**Figure S2.** Sensitivity analysis showing the robust association between LHR and MAFLD in PLWH. MSM, men who have sex with men; ART, antiretroviral therapy; NNRTIs, non-nucleoside reverse transcriptase inhibitors; INSTIs, integrase strand transfer inhibitors; PIs, protease inhibitors; 3TC, lamivudine; DTG, dolutegravir; HDL-C, high-density lipoprotein cholesterol; TG, triglycerides.


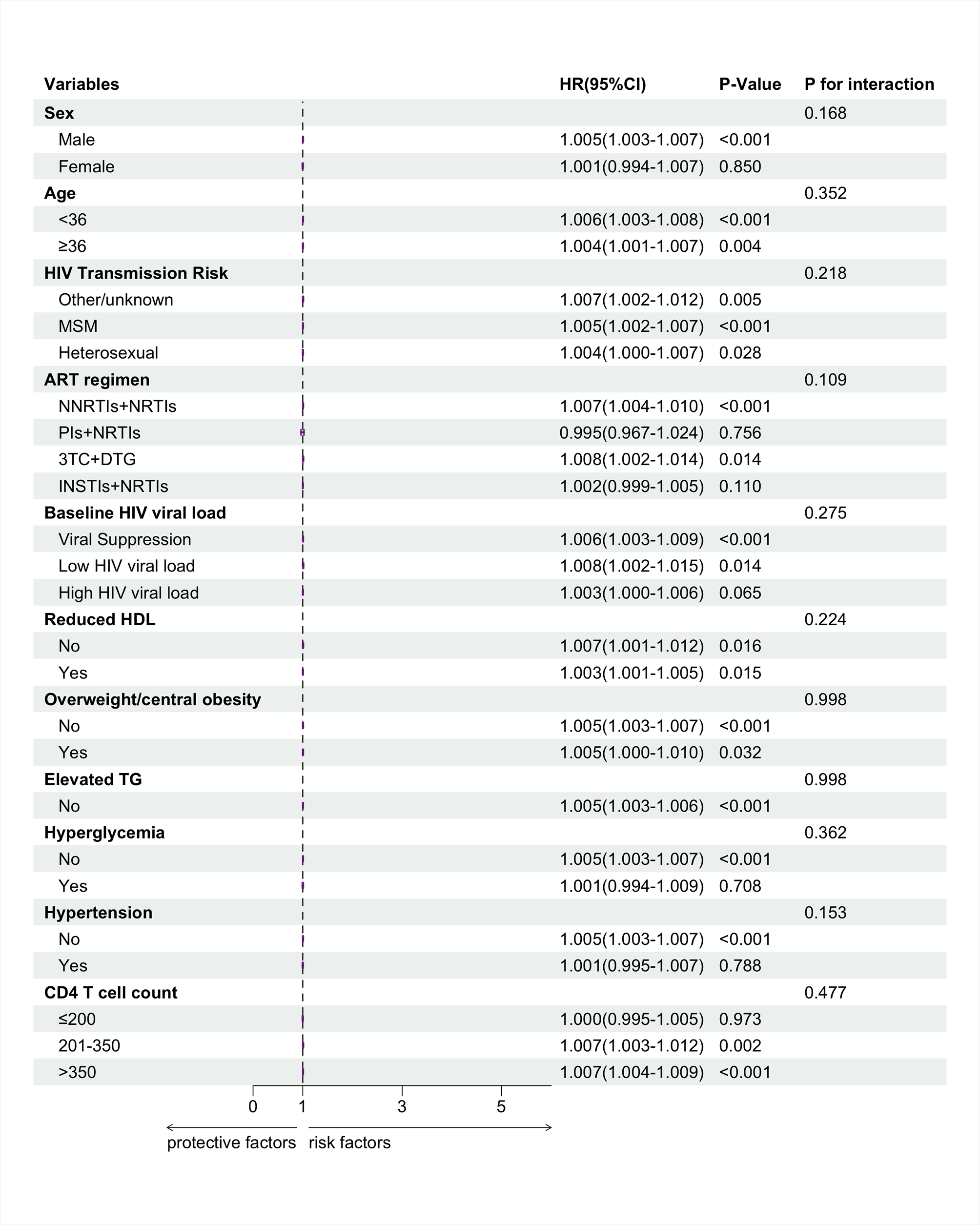
 **Figure S3.** Sensitivity analysis showing the robust association between PHR and MAFLD in PLWH. MSM, men who have sex with men; ART, antiretroviral therapy; NNRTIs, non-nucleoside reverse transcriptase inhibitors; INSTIs, integrase strand transfer inhibitors; PIs, protease inhibitors; 3TC, lamivudine; DTG, dolutegravir; HDL-C, high-density lipoprotein cholesterol; TG, triglycerides.


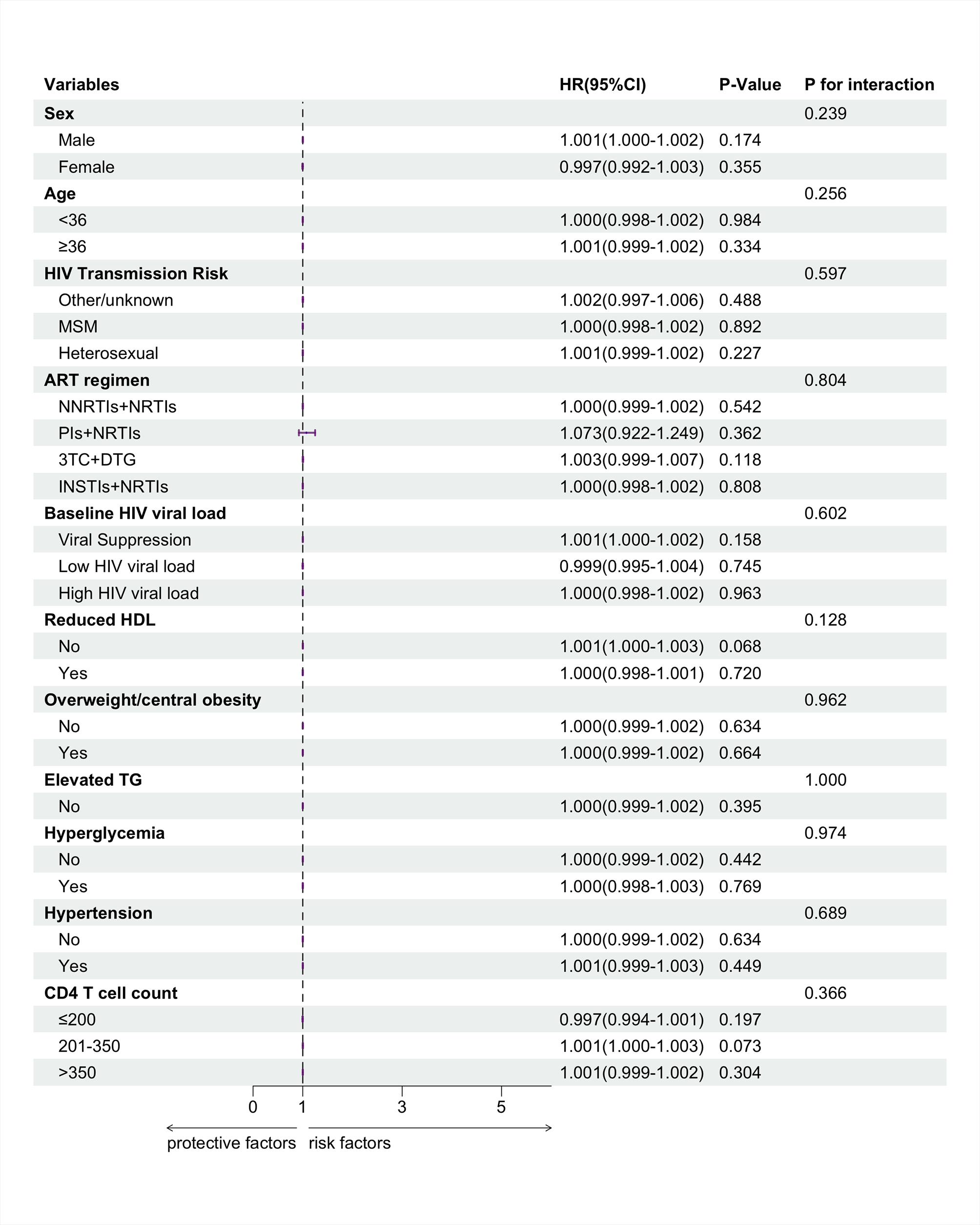
 **Figure S4.** Sensitivity analysis showing the robust association between AISI and MAFLD in PLWH. MSM, men who have sex with men; ART, antiretroviral therapy; NNRTIs, non-nucleoside reverse transcriptase inhibitors; INSTIs, integrase strand transfer inhibitors; PIs, protease inhibitors; 3TC, lamivudine; DTG, dolutegravir; HDL-C, high-density lipoprotein cholesterol; TG, triglycerides.
